# Clinical, genomic, and epigenomic analyses of H3K27M-mutant diffuse midline glioma long-term survivors reveal a distinct group of tumors with MAPK pathway alterations

**DOI:** 10.1007/s00401-023-02640-7

**Published:** 2023-10-18

**Authors:** Holly J. Roberts, Sunjong Ji, Alberto Picca, Marc Sanson, Mekka Garcia, Matija Snuderl, Ulrich Schüller, Thiébaud Picart, François Ducray, Adam L. Green, Yoshiko Nakano, Dominik Sturm, Zied Abdullaev, Kenneth Aldape, Derek Dang, Chandan Kumar-Sinha, Yi-Mi Wu, Dan Robinson, Josh N. Vo, Arul M. Chinnaiyan, Rodrigo Cartaxo, Santhosh A. Upadhyaya, Rajen Mody, Jason Chiang, Suzanne Baker, David Solomon, Sriram Venneti, Drew Pratt, Sebastian M. Waszak, Carl Koschmann

**Affiliations:** 1 Department of Pediatrics, Michigan Medicine, Ann Arbor, MI USA; 2 Department of Neurology-2, Pitié-Salpêtrière University Hospital, Assistance Publique-Hôpitaux de Paris (AP-HP), Paris, France; 3 Onconeurotek, AP-HP, Hôpital Pitié-Salpêtrière, 75013 Paris, France; 4 Sorbonne Université, Inserm, CNRS, UMR S 1127, Institut du Cerveau et de la Moelle Épinière, ICM, Paris, France; 5 Department of Neurology, NYU Langone Health, New York, NY USA; 6 Department of Pathology, NYU Langone Health, New York, NY USA; 7 Research Institute Children’s Cancer Center Hamburg, Hamburg, Germany; 8 Department of Pediatric Hematology and Oncology, University Medical Center Hamburg-Eppendorf, Hamburg, Germany; 9 Institute of Neuropathology, University Medical Center Hamburg-Eppendorf, Hamburg, Germany; 10 Department of Neurosurgical Oncology and Vascular Neurosurgery, Pierre Wertheimer Neurological and Neurosurgical Hospital, Hospices Civils de Lyon, Université Lyon 1, CRCL, UMR Inserm 1052, CNRS 5286, 69008 Lyon, France; 11 Neuro-Oncology Department, Hospices Civils de Lyon, Université Lyon 1, CRCL, UMR Inserm 1052, CNRS 5286, 69000 Lyon, France; 12 Morgan Adams Foundation Pediatric Brain Tumor Research Program, Department of Pediatrics, University of Colorado School of Medicine, Aurora, CO USA; 13 Division of Brain Tumor Translational Research, National Cancer Center Research Institute, 5-1-1, Tsukiji, Chuo-ku, Tokyo, 104-0045 Japan; 14 Department of Pediatric Hematology/Oncology, Osaka City General Hospital, Osaka, Japan; 15 Hopp Children’s Cancer Center Heidelberg (KiTZ), Heidelberg, Germany; 16 Department of Pediatric Hematology, Oncology, Immunology and Pulmonology, Heidelberg University Hospital, Heidelberg, Germany; 17 Division of Pediatric Glioma Research, German Cancer Research Center (DKFZ) and German Consortium for Translational Cancer Research (DKTK), Heidelberg, Germany; 18 Laboratory of Pathology, Center for Cancer Research, National Cancer Institute, National Institutes of Health, Bethesda, MD USA; 19 Department of Pathology, University of Michigan Medical School, Ann Arbor, MI USA; 20 Michigan Center for Translational Pathology, University of Michigan, Ann Arbor, MI USA; 21 Rogel Cancer Center, University of Michigan, Ann Arbor, MI USA; 22 Howard Hughes Medical Institute, University of Michigan, Ann Arbor, MI USA; 23 Department of Urology, University of Michigan, Ann Arbor, MI USA; 24 Department of Computational Medicine and Bioinformatics, University of Michigan, Ann Arbor, MI USA; 25 Department of Pathology, St. Jude Children’s Research Hospital, Memphis, TN USA; 26 Department of Developmental Neurobiology, St. Jude Children’s Research Hospital, Memphis, TN USA; 27 Department of Pathology, University of California, San Francisco, San Francisco, CA USA; 28 Laboratory of Computational Neuro-Oncology, School of Life Sciences, Swiss Institute for Experimental Cancer Research, École Polytechnique Fédérale de Lausanne (EPFL), Lausanne, Switzerland; 29 Department of Neurology, University of California, San Francisco, San Francisco, CA USA; 30 EPFL SV ISREC UPWASZAK, AAB 238 (Batiment AAB), Station 19, 1015 Lausanne, Switzerland; 31 University of Michigan, 3520D MSRB I, 1150 W Medical Center Dr, Ann Arbor, MI 48109 USA

Diffuse midline gliomas (DMGs) harboring loss of H3K27me3 due to H3K27M/I mutations or *EZHIP* overexpression comprise a World Health Organization (WHO) defined subtype of pediatric diffuse high-grade gliomas (H3K27-altered DMG), which carries a poor prognosis with median overall survival (OS) of 12 months [2, 3]. H3K27M-mutant DMGs (H3K27M-DMGs) additionally develop somatic alterations in driver genes (eg, *TP53, PPM1D, ATRX, PIK3CA, ACVR1*) during tumor evolution. DNA methylation profiling of H3K27M-DMGs reveals distinct clustering in comparison to other pediatric-type diffuse high-grade gliomas. Despite the generally poor prognosis, long-term survivors (LTS) have been reported with distinct molecular profiles such as somatic *FGFR1* mutations [6] and rare cases with non-diffuse (circumscribed) histology patterns [4]. Here, we aim to identify clinical, genomic, and epigenomic characteristics of LTS patients with H3K27M-DMG.

Through a comprehensive multi-site and literature case review, we identified 85 patients with confirmed H3K27M-DMG (by DNA sequencing or IHC) and LTS, defined as OS of at least 36 months from initial diagnosis (Online Resource [OR] Table S1). We extracted available demographic, diagnostic, genomic, therapeutic, and clinical outcome data and performed DNA methylation analysis when tissue was available (30 samples from 26 patients). A control cohort of 453 patients with confirmed H3K27M-DMG and OS < 18 months (short-term survival [STS]) with detailed histology, demographic, and CNS tumor location from Pratt et al. [4] was utilized. We further assembled two molecular cohorts with 258 H3K27M-DMG patients (208 STS patients) with clinical and genomic profiles (see OR) and 20 STS H3K27M-DMG patients (MNP2.0 cohort) with clinical, genomic, and tumor DNA methylation profiles.

The median age of our LTS cohort was 13.2 years (interquartile range [IQR] 7–30 years), 64.7% of patients were females, and the median OS was 51.6 months (IQR 40.7–63.8 months). Clinically, patients received a variety of therapy types of various durations and timing throughout disease courses (Fig. 1a). At diagnosis, LTS patients had a greater frequency of non-infiltrative/circumscribed tumors (18.7% *vs* 1.7%, *P* < 0.0001) and were more frequently diagnosed with tumors in the thalamus (42.3% *vs* 14.7%, *P* < 0.0001) compared to STS patients. No statistical association was found between H3.1 *vs* H3.3K27M and survival (12.3% STS *vs* 14.2% LTS with H3.1, *P* = 0.85; OR Fig. S1).

**Fig. 1:**
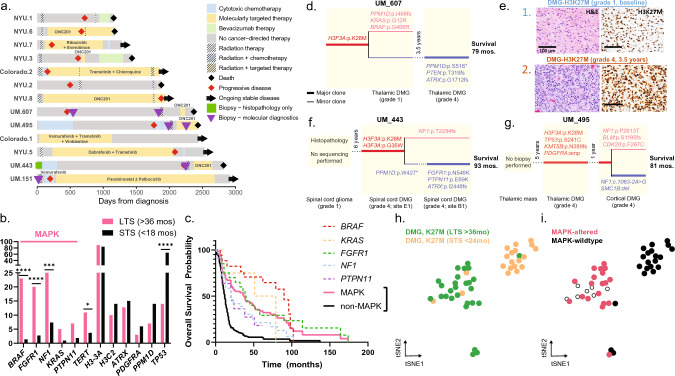
Characteristics of LTS patients. **a** Swimmer plot for 13 LTS patients demonstrates wide variety in timing and types of therapies. **b** Bar plot comparing oncogene mutation frequencies in LTS (n = 55) and molecular control (OS < 18 months, n = 208) cohorts highlights the higher frequency of MAPK pathway alterations in the LTS cohort. **c** Kaplan–Meier curve for combined LTS and molecular control cohort (n = 310) reveals that MAPK pathway genetic alterations are associated with improved OS at 36 months and shows differential survival among MAPK oncogenes. **d** Clonal map demonstrates molecular evolution of UM-607 from time of initial and subsequent biopsies, with thin lines for sub-clonal (< 10%) and thick lines for clonal branching events. **e** Histology stains demonstrate histologic evolution from low- to high-grade. **f–g** Clonal maps demonstrate the molecular evolution of UM-443 and UM-495 at various timepoints and tumor samples. **h–i** Joint analysis using *t*-SNE dimensionality reduction of LTS (23 patients, 27 tumors) and STS (20 patients; MNP2.0 cohort) H3K27M-DMGs reveals two clusters separating tumors from LTS/STS patients (**h**) and tumors with/without MAPK pathway alterations (**i**)

Evaluation of the genomic landscape of LTS patients (OR Fig. S2) revealed a high frequency of MAPK pathway alterations (69.0%, 38/55) compared to STS patients (12.0%, 25/208, *P* < 0.001). Clonality of mutations is included for five patients with available variant allele frequency (VAF) data (sub-clonal defined as VAF < 10%) in OR Table S2. The most frequently mutated MAPK pathway genes in LTS patients were *BRAF* (23.6%, n = 13/55), *NF1* (23.6%, n = 13/55), and *FGFR1* (21.8%, n = 12/55; Fig. 1b, OR Table S1). In addition, seven LTS patients presented with MAPK-associated mutations in *PTPN11* (9.1%, n = 5/55) and *KRAS* (5.5%, n = 3/55). In contrast, individual MAPK genes were rarely mutated in STS patients (7.7% *NF1*, 2.4% *FGFR1*, 1.4% *BRAF*, 1.4% *PTPN11*, 1.0% *KRAS*). Moreover, we observed a striking absence of somatic *TP53* mutations in LTS patients (16.4%, 9/55 *vs* 65.4%, 136/208, *P* < 0.0001). A combined survival analysis of our LTS and molecular control cohort (n = 310) revealed that MAPK pathway alterations are associated with LTS (MAPK-altered *vs* wildtype, OS at 36 months 51.3% *vs* 7.6%; Fig. 1c). A univariate and multivariate (sex, age, H3.1/H3.3, *TP53,* MAPK) survival analysis that was restricted to our molecular control cohort (n = 258 patients) further demonstrated that MAPK pathway alterations are associated with LTS (OS at 36 months 7.4% *vs* 0%; OR Figs. S3, S4). This combined retrospective molecular cohort may be a closer reflection of the prevalence of patients with LTS and MAPK alterations in the general H3K27M-DMG population (5–10%).

We further investigated the tumor evolution of three cases with multiple biopsies for histology review and molecular profiling (Fig. 1d–g). UM-607 was diagnosed with a tectal-thalamic H3K27M-DMG with diffuse, low-grade histology (see Fig. 1d inlay “1”, Fig. 1e) but developed progression 3.5 years later, and re-biopsy showed high-grade features (Fig. 1d inlay “2”, Fig. 1e) and a significantly altered tumor genomic profile, ultimately surviving 79 months from diagnosis. UM-443 had a spinal cord glioma initially diagnosed as low-grade glioma (LGG) on histology review with no tumor sequencing performed, and later was interpreted as high-grade on histology and with H3K27M mutation with intra-tumoral genomic heterogeneity as evidenced by phylogenetic analysis (Fig. 1f and OR Fig. S5). UM-495 presented with a thalamic mass, presumed to be LGG on imaging so biopsy was deferred; however, biopsy five years later confirmed H3K27M-DMG, and subsequent biopsy one year later of a cortical metastasis revealed additional molecular alterations (Fig. 1g). As these phylogenetic trees demonstrate, H3K27M mutations were the founding events in all patients and MAPK alterations were either clonal or sub-clonal. Also, both patients (UM-607, UM-443) with initial low-grade histology lacked *TP53* mutations.

Finally, DNA methylation-based classification of LTS DMGs (26 patients; n = 30 tumors) against 40 reference gliomas, glioneuronal tumors, and neuronal tumors described by Capper et al. [1] showed closest association with H3K27M-DMGs (OR Fig. S6). Three LTS samples mapped closest to healthy brain tissues and were presumably of low tumor content. We next performed a joint analysis of LTS (n = 23 patients) and STS H3K27M-DMG samples (n = 20 patients, MNP2.0 cohort) and discovered two clusters that separate LTS and STS patients (Fig. 1h). Strikingly, MAPK pathway mutations were exclusively seen in the LTS methylation cluster (Fig. 1i). The LTS cluster included three STS cases, all with MAPK alterations. This data suggests that the LTS DNA methylation cluster is potentially defined by MAPK alterations. DNA methylation of multi-timepoint LTS samples from UM cases revealed that all map into the MAPK H3K27M-DMG cluster (OR Fig. S7).

In summary, we identify enrichment of alterations in MAPK pathway genes in patients with LTS compared to those with typical survival. Furthermore, H3K27M-DMGs with MAPK alterations demonstrate a unique DNA methylation signature, even in some cases without a MAPK in initial sample, thus raising the possibility of a unique evolutionary trajectory that selects for subsequent MAPK alteration (eg, UM-443 and UM-495). The current WHO classification includes the entity “diffuse low-grade glioma, MAPK pathway-altered”, which is defined by genetic alterations in *BRAF* and *FGFR1*. Moreover, circumscribed astrocytic gliomas include “high-grade astrocytomas with piloid features” with recurrent MAPK (*NF1, FGFR1*) alterations [3, 5]. Our data demonstrates distinct clinical outcomes, DNA methylation patterns, MAPK mutations, and absence of *TP53* mutations that supports a distinct LTS-associated subtype of H3K27M-DMG, which we propose as “diffuse midline glioma, H3K27M-mutant and MAPK pathway-altered”. As a few MAPK-wildtype cases clustered with the “MAPK-altered” group, methylation analysis should be considered with other clinical and molecular diagnostic elements. Our study also confirms that a subset of H3K27M-DMGs show circumscribed histology, and this contradiction with the diagnostic category “diffuse” midline glioma may need to be re-addressed in the future.

Limitations of our study include a relatively small sample of patients with LTS and clinical data limited by availability in the literature in many cases. Further investigation into the biology of H3K27M/MAPK-altered tumors is warranted to confirm our findings. Overall, our results suggest that numerous factors contribute to LTS in patients with H3K27M-DMG, particularly low-grade and non-infiltrative histology, thalamic/non-brainstem location, absence of somatic *TP53* mutations, and enrichment of MAPK pathway alterations. These findings further support the use of biopsy in these patients for clarification of H3K27M-DMG subtype, prognosis, and treatment.

### Supplementary Information

Below is the link to the electronic supplementary material.

Supplementary file 1 (PDF 2694 kb)

Supplementary file 2 (XLSX 16 kb)

Supplementary file 3 (XLSX 9 kb)

## Data Availability

All tumor somatic sequencing data generated from MiOncoSeq has been uploaded to the Database of Genotypes and Phenotypes (dbGaP) [accession number phs000673.v1.p1]. For all other patients, genetic results were used as reported by the literature or treating physician. Publicly available data that support the findings of this study were obtained from Pratt et al. [4] and the MNP2.0 study. All other data generated in this study are available upon reasonable request.
