# Supplementary material for: Clinical, genomic, and epigenomic analyses of H3K27M-mutant diffuse midline glioma long-term survivors reveal a distinct group of tumors with MAPK pathway alterations
Source: Acta Neuropathol. 2023 Oct 18;146(6):849–52. doi: 10.1007/s00401-023-02640-7 (PMC10627895; doi:10.1007/s00401-023-02640-7)
Supplement: Supplementary file 1 — (PDF 2694 kb) [file 401_2023_2640_MOESM1_ESM.pdf]

Online Resource

Acta Neuropathologica

Clinical, genomic, and epigenomic analyses of H3K27M-mutant diffuse midline glioma long-term survivors reveal a distinct group of tumors with MAPK pathway alterations

Holly J. Roberts[1^], Sunjong Ji[1^], Alberto Picca[2], Marc Sanson[2, 3, 4], Mekka Garcia[5], Matija Snuderl[6], Ulrich Schüller[7, 8, 9], Thiébaud Picart[10], François Ducray[11], Adam L. Green[12], Yoshiko Nakano[13, 14], Dominik Sturm[15, 16, 17], Zied Abdullaev[18], Kenneth Aldape[18], Derek Dang[19], Chandan Kumar-Sinha[19, 20], Yi-Mi Wu[19, 20], Dan Robinson[19, 20], Josh N. Vo[19, 20], Arul M. Chinnaiyan[19, 20, 21, 22, 23, 24], Rodrigo Cartaxo[1], Santhosh A. Upadhyaya[1], Rajen Mody[1], Jason Chiang[25], Suzanne Baker[26], David Solomon[27], Sriram Venneti[1, 19], Drew Pratt[18], Sebastian M. Waszak[28,29*^], Carl Koschmann[1*^]

1. Department of Pediatrics, Michigan Medicine, Ann Arbor, MI, USA.
2. Department of Neurology-2, Pitié-Salpêtrière University Hospital, Assistance Publique-Hôpitaux de Paris (AP-HP), Paris, France
3. Onconeurotek, AP-HP, Hôpital Pitié-Salpêtrière, F-75013, Paris, France.
4. Sorbonne Université, Inserm, CNRS, UMR S 1127, Institut du Cerveau et de la Moelle épinière, ICM, Paris, France.
5. Department of Neurology, NYU Langone Health, New York, New York, USA.
6. Department of Pathology, NYU Langone Health, New York, New York, USA.
7. Research Institute Children's Cancer Center Hamburg, Hamburg, Germany.
8. Department of Pediatric Hematology and Oncology, University Medical Center Hamburg-Eppendorf, Hamburg, Germany.
9. Institute of Neuropathology, University Medical Center Hamburg-Eppendorf, Hamburg, Germany.
10. Department of Neurosurgical Oncology and Vascular Neurosurgery, Pierre Wertheimer Neurological and Neurosurgical Hospital, Hospices Civils de Lyon, Université Lyon 1, CRCL, UMR Inserm 1052_CNRS 5286, 69008 Lyon, France.
11. Neuro-Oncology Department, Hospices Civils de Lyon, Université Lyon 1, CRCL, UMR Inserm 1052_CNRS 5286, 69000 Lyon, France.
12. Morgan Adams Foundation Pediatric Brain Tumor Research Program, Department of Pediatrics, University of Colorado School of Medicine, Aurora, CO, USA.
13. Division of Brain Tumor Translational Research, National Cancer Center Research Institute, 5-1-1, Tsukiji, Chuo-ku, Tokyo, 104-0045, Japan. yonakano@ncc.go.jp.
14. Department of Pediatric Hematology/Oncology, Osaka City General Hospital, Osaka, Japan. yonakano@ncc.go.jp.
15. Hopp Children's Cancer Center Heidelberg (KiTZ), Heidelberg, Germany
16. Department of Pediatric Hematology, Oncology, Immunology and Pulmonology, Heidelberg University Hospital, Heidelberg, Germany
17. Division of Pediatric Glioma Research, German Cancer Research Center (DKFZ) and German Consortium for Translational Cancer Research (DKTK), Heidelberg, Germany.
18. Laboratory of Pathology, Center for Cancer Research, National Cancer Institute, National Institutes of Health, Bethesda, MD, USA.

19. Department of Pathology, University of Michigan Medical School, Ann Arbor, MI, USA.
20. Michigan Center for Translational Pathology, University of Michigan, Ann Arbor, MI, USA.
21. Rogel Cancer Center, University of Michigan, Ann Arbor, MI, USA.
22. Howard Hughes Medical Institute, University of Michigan, Ann Arbor, MI, USA.
23. Department of Urology, University of Michigan, Ann Arbor, MI, USA.
24. Department of Computational Medicine and Bioinformatics, University of Michigan, Ann Arbor, MI, USA.
25. Department of Pathology, St. Jude Children's Research Hospital, Memphis, TN, USA.
26. Department of Developmental Neurobiology, St. Jude Children's Research Hospital, Memphis, TN, USA.
27. Department of Pathology, University of California, San Francisco, San Francisco, CA, USA.
28. Laboratory of Computational Neuro-Oncology, Swiss Institute for Experimental Cancer Research, School of Life Sciences, École Polytechnique Fédérale de Lausanne (EPFL), Lausanne, Switzerland.
29. Department of Neurology, University of California, San Francisco, San Francisco, CA, USA.

^ contributed equally to this work
*Correspondence to:

1. Carl Koschmann, MD
   3520D MSRB I
   1150 W Medical Center Dr
   University of Michigan
   Ann Arbor MI, 48109
   Office (p) 734-615-2736 (f) 734-763-2543
   ckoschma@med.umich.edu

2. Sebastian M. Waszak, PhD
   EPFL SV ISREC UPWASZAK
   AAB 238 (Batiment AAB)
   Station 19
   CH-1015 Lausanne
   sebastian.waszak@epfl.ch
   +41 21 693 6806

Table of Contents

Materials and Methods	Page 1-2
Supplementary Figures	
Figure S1	Page 3
Figure S2	Page 4
Figure S3	Page 5
Figure S4	Page 6
Figure S5	Page 7
Figure S6	Page 8
Figure S7	Page 9
Table S1 (ESM_2)	Separate supplemental file
Table S2 (ESM_3)	Separate supplemental file
Supplementary References	Page 10

Materials and Methods

Ethics approval

This retrospective study was approved by the University of Michigan Medical School Institutional Review Board (IRBMED). HIPAA authorization for use of previously collected de-identified data was waived by IRBMED.

Comparison of characteristics between LTS and STS cohorts

A control cohort of 453 patients with confirmed H3K27M-DMG and OS < 18 months from Pratt, et al [4] with detailed histology, demographic and CNS tumor location with the LTS cohort for statistically significance (Two-sided Fisher's exact test), excluding unknown variables. Frequent genetic alterations were separately compared between patients in the LTS cohort and 208 H3K27M-DMG patients with genomic and clinical data from PBTA, PNOC003, HERBY II, and ICR London cohorts for statistical significance (Two-sided Fisher's exact test). Differences in overall survival based on presence of genetic alterations within the MAPK pathway were analyzed for 310 patients from the LTS and molecular control cohorts using the log-rank test using GraphPad Prism software (version 9). This was then repeated using only the molecular control cohort (n=258). Multivariate survival analysis was based on cox proportional hazards model (R survival package v3.5; coxph function) and visualized using forest plots (R package survminer v0.4.9; ggforest function).

Methylation

DNA methylation profiling with Illumina EPIC and 450k arrays was performed for 26 patients, including multiple timepoints for three patients for a total of 30 tumors. A comparison cohort of H3K27M-DMGs short-term survivors (OS<18 months; n=20) with genetically confirmed H3K27M mutations and DNA methylation data and DNA panel sequencing data was obtained from the MNP2.0 study and was utilized for comparison within H3 K27M DMG LTS tumors [3].

Raw IDAT files from LTS H3K27M DMG samples (n=30; this study), STS H3K27M DMG samples (n=20; [3]), and reference glioma, glioneuronal tumors, and neuronal tumor samples (40 tumor classes and nine control brain tissue classes; ref. [1]; max. 30 samples per tumor class) were processed with the R package RnBeads (v2.16; default settings). Samples processed with the EPIC and 450k array technology were merged using the rnb.combine.arrays function and common probes. The 20,000 most variable autosomal probes were used for tSNE analysis of 1,010 LTS tumors, reference gliomas, glioneuronal tumors, neuronal tumor, and control brain tissue samples (Rtsne package v0.16; perplexity = 30, iterations = 5000). The 10,000 most variable autosomal probes were used for tSNE analysis of 47 LTS and STS H3K27M DMG samples (perplexity = 5, iterations = 5000). DNA methylation-based classification of LTS H3K27M DMG samples was based on the Heidelberg brain tumor classifier (v12.5; https://www.molecularneuropathology.org/mnp/).

Genetic sequencing

Pediatric patients who were seen at the University of Michigan (UM) were offered enrollment in the IRB-approved MiOncoSeq study for next-generation DNA (1,700 gene panel) using previously established methods [1, 2]. All tumor somatic sequencing data generated from MiOncoSeq has been uploaded to the Database of Genotypes and Phenotypes (dbGaP) [accession number

phs000673.v1.p1]. Somatic mutations were processed using vcf2maf (v1.6.19) and alterations in known pHGG/DMG driver genes were visualized using the R package ComplexHeatmap (v2.13.1). For all other patients, genetic results were used as reported by the literature or treating physician.

Supplementary Figures

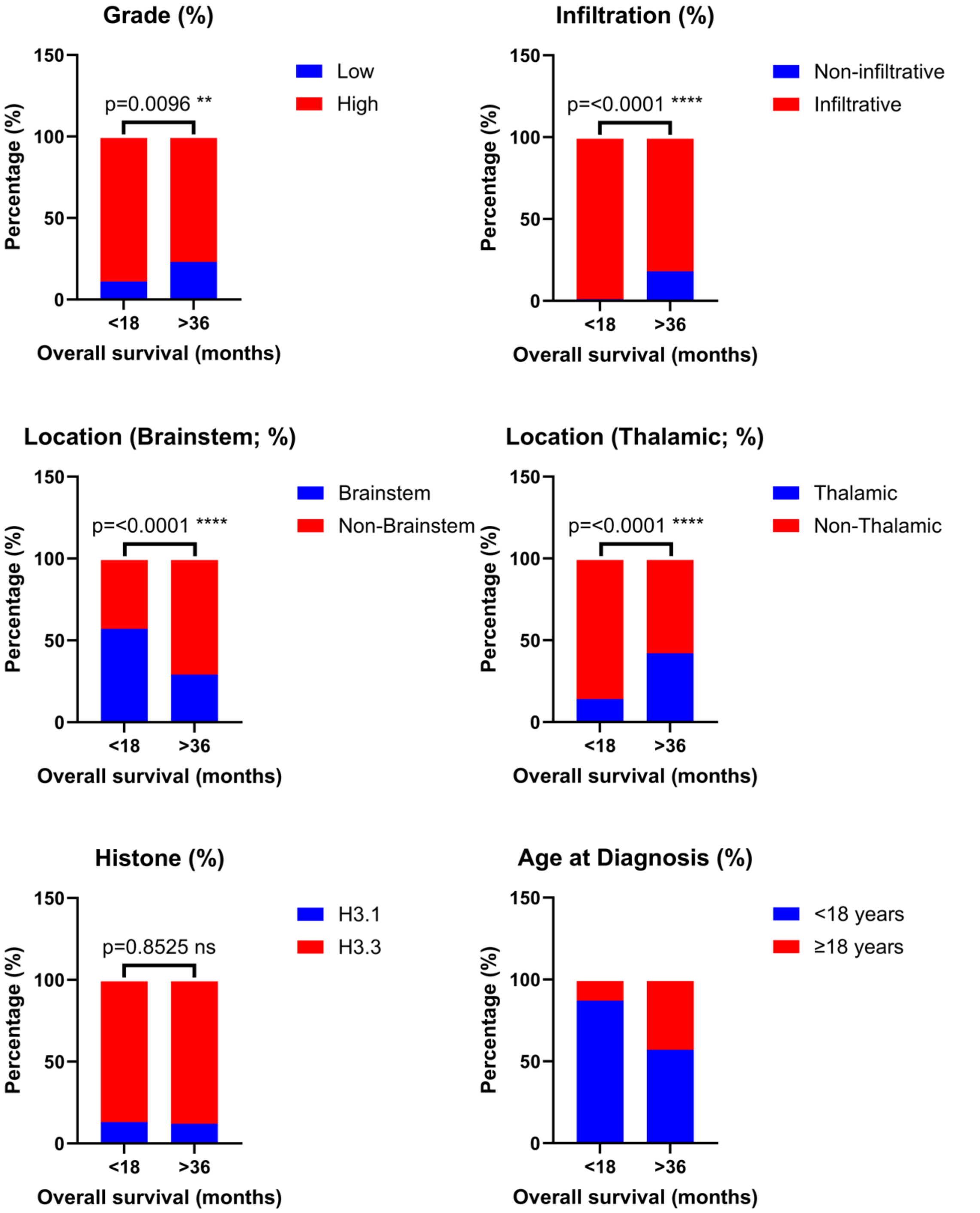

Figure S1. Bar plots compare patient characteristics between our LTS cohort and STS cohort derived from Pratt, et al (n=453). LTS cohort has significantly higher frequency of low-grade histology, non-infiltrative/circumscribed histology, and thalamic location as well as lower frequency of brainstem location compared to the STS cohort. Statistical analysis deferred for age at diagnosis between cohorts given this is a continuous variable though shown for comparison.

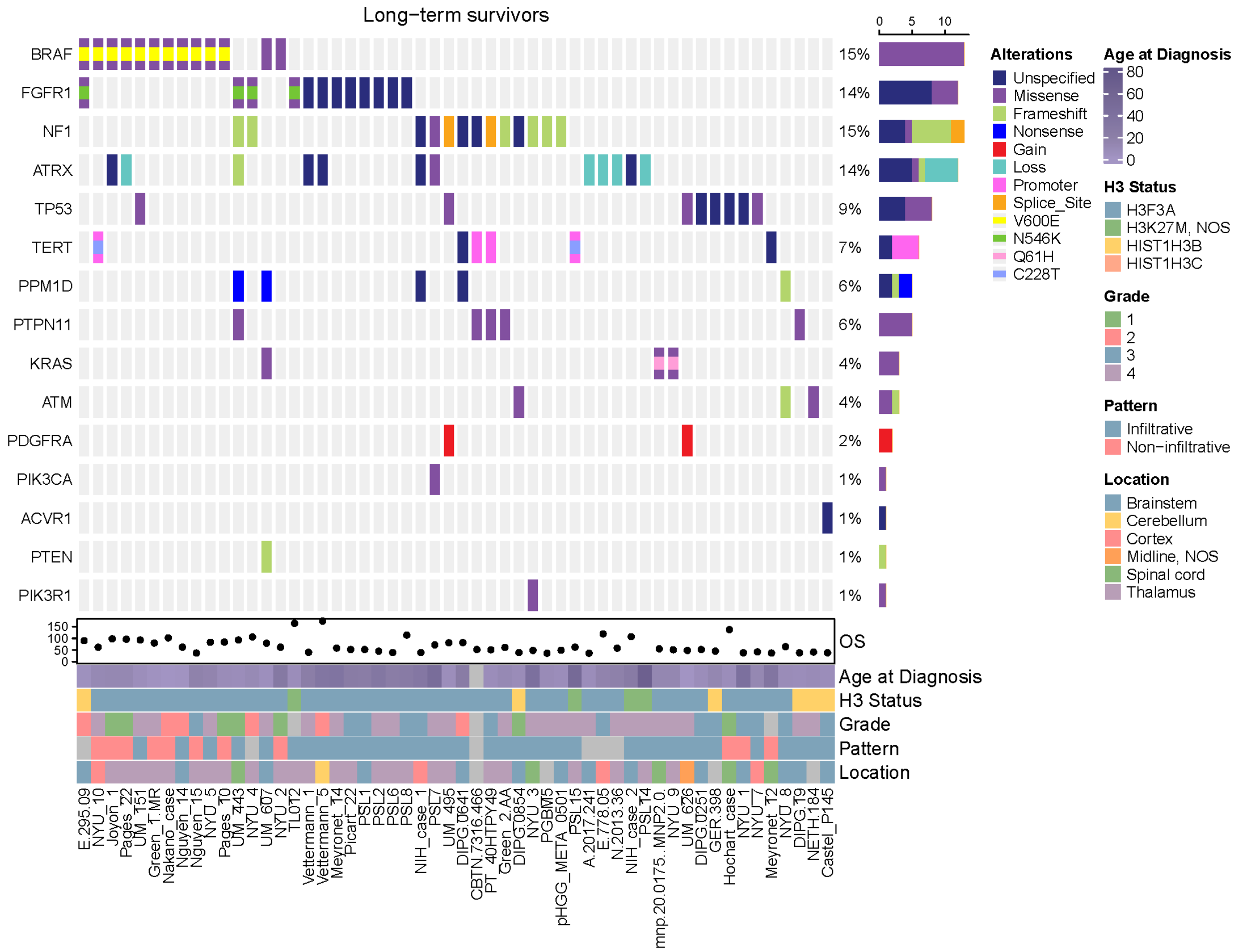

Figure S2. OncoPrint depicts genomic alterations in addition to OS, age, tumor grade, histology pattern, and tumor location in LTS cohort (n=55), highlighting the high prevalence of genetic alterations in MAPK-related genes in this cohort.

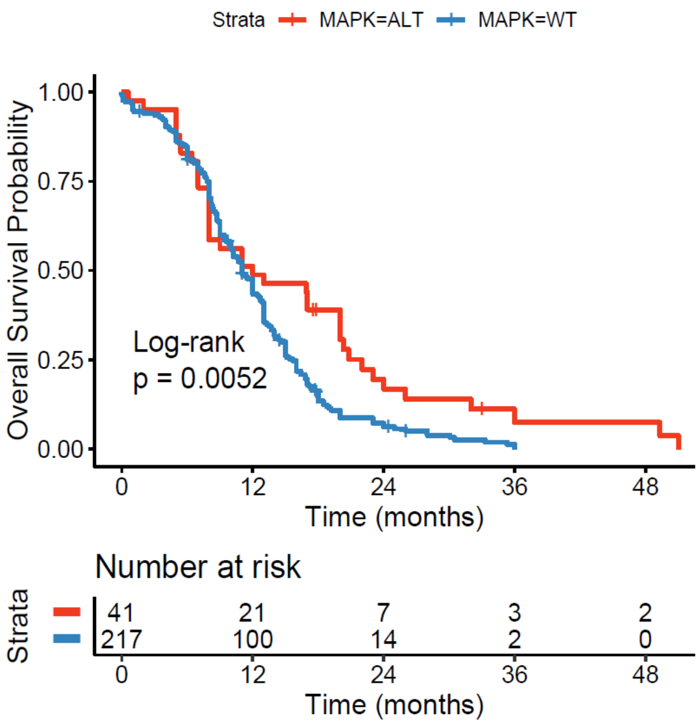

Figure S3. Kaplan-Meier curve demonstrates significantly improved OS in 258 patients from our molecular control cohort with alterations in MAPK genes (*NF1, PTPN11, FGFR1, BRAF, KRAS*) compared to those with MAPK wt.

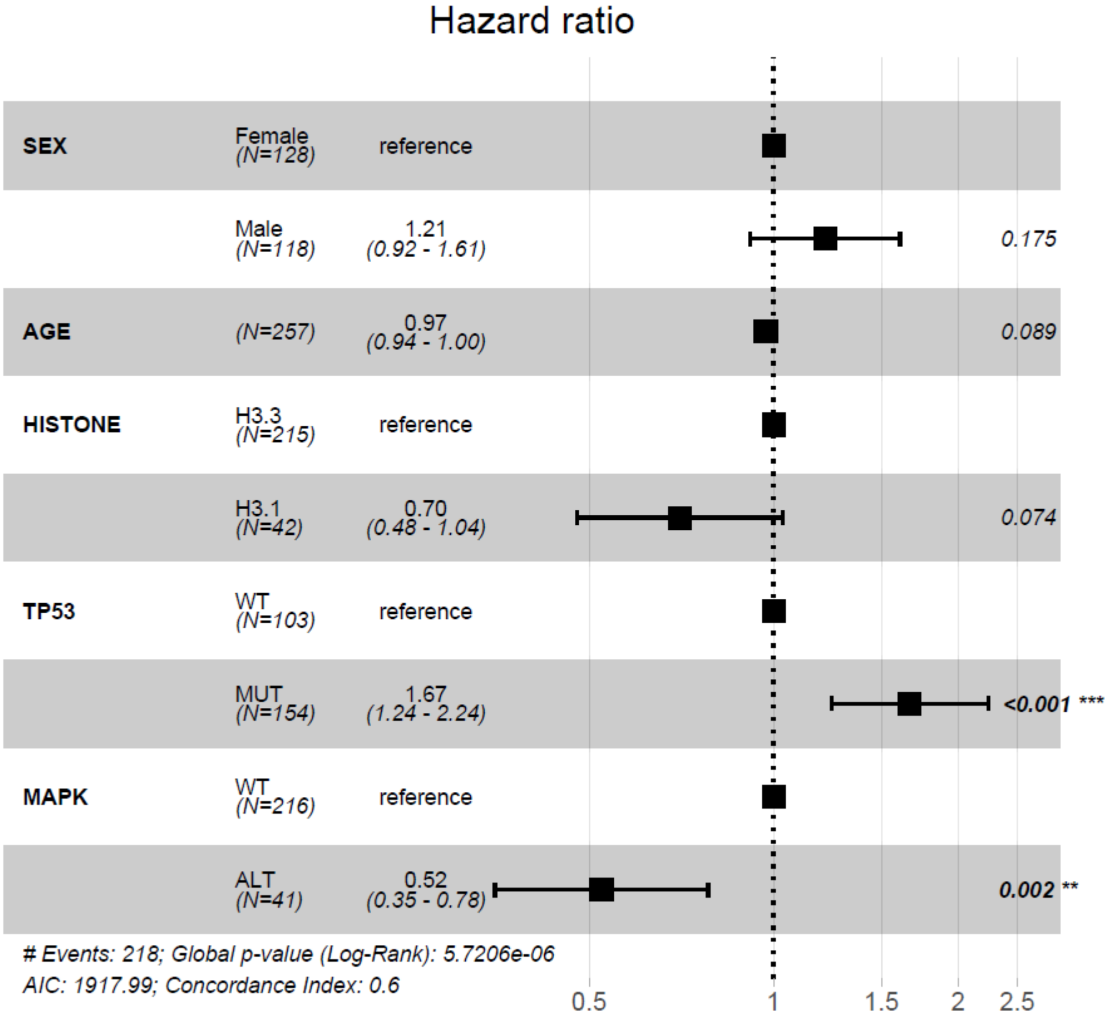

Figure S4 Multivariate analysis reveals that the presence of MAPK alteration is associated with LTS, independent of sex, age, histone mutation, or *TP53* mutation status. *TP53* also remains associated with STS independent of these variables.

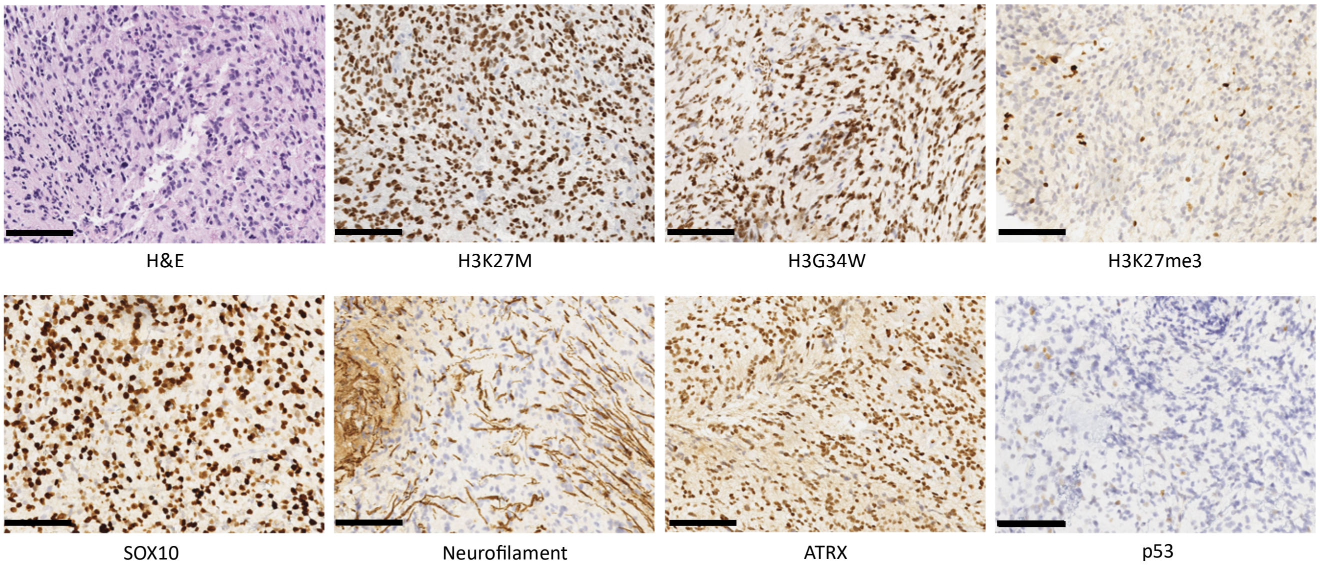

Figure S5. Representative images of histology slides for UM-443 at diagnosis captured at 40x magnification from H&E, H3K27M, H3G34W, H3K27me3, SOX10, Neurofilament, ATRX, and p53 immunostaining. Scale bar in lower left corner indicates 100 μm.

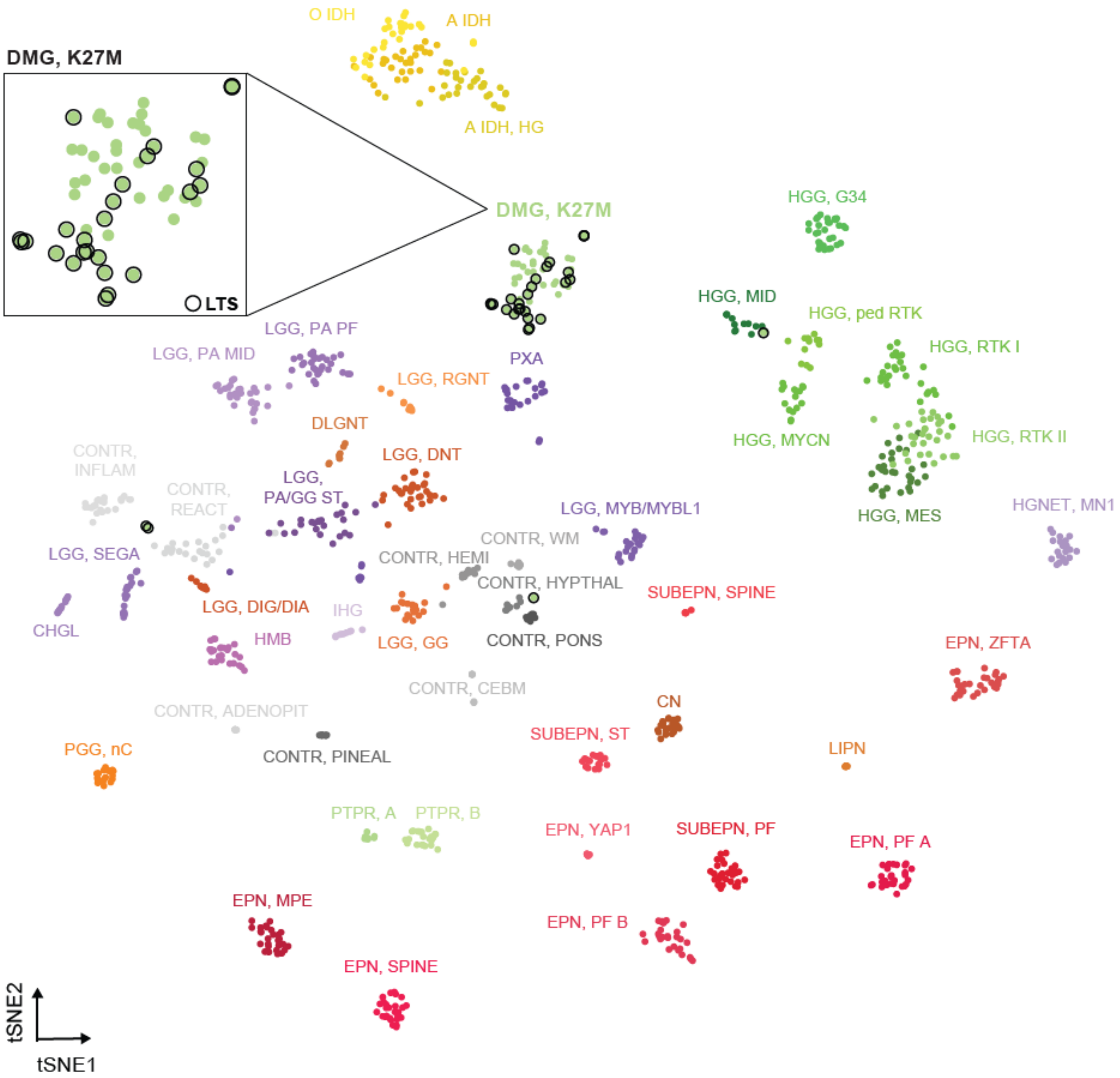

Figure S6. DNA methylation classification of LTS DMGs (n=30) against 40 reference glioma, glioneuronal, and neuronal tumor entities on *t*-SNE map reveals that these tumors mostly cluster within DMG, K27M classification. Three LTS DMGs cluster near normal brain tissue, likely reflecting low tumor content of the sample, and one clusters with HGG, MID.

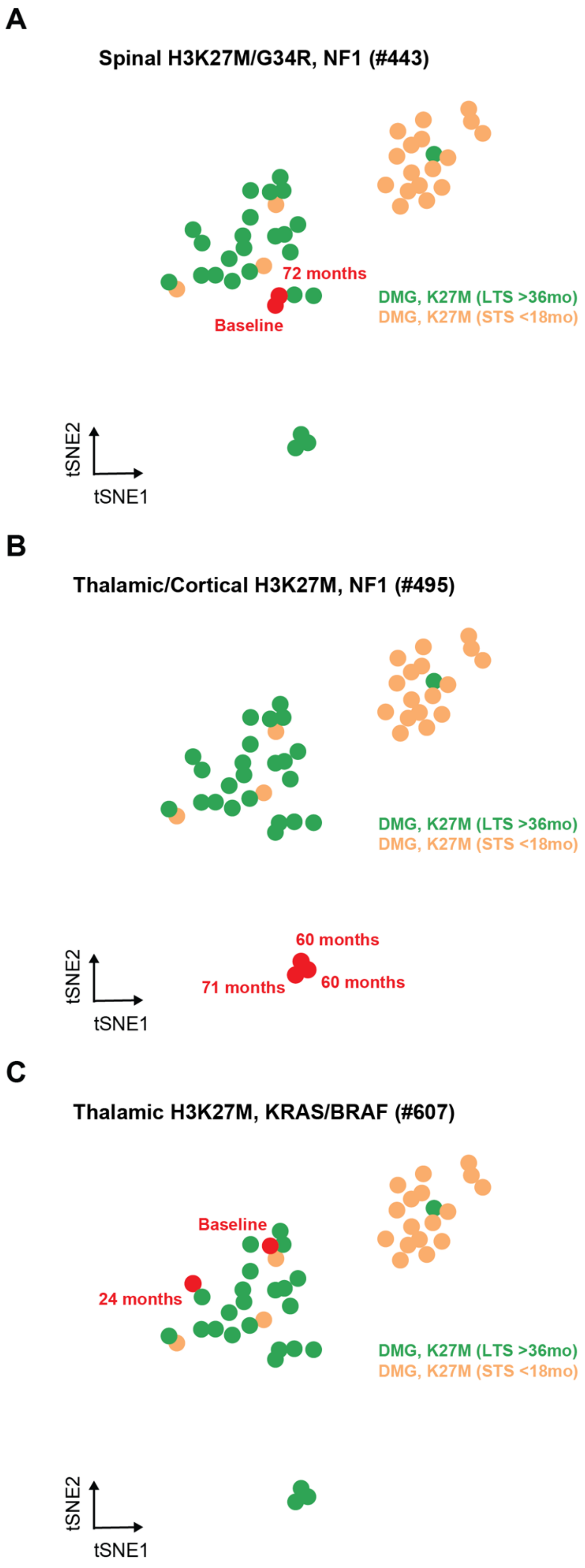

Figure S7. *t*-SNE map of LTS (green) and STS (orange) cohorts. Each *t*-SNE map highlights tumor samples from the same patient (UM-443, UM-495, and UM-607) in red with timing of tumor sampling from diagnosis, which demonstrates that despite evolution of genetic mutations, methylation profiling remains relatively stable throughout the course of tumor evolution. Importantly, at all timepoints, these tumors all map into the MAPK DMG cluster as seen in **Fig. 1i.**

Supplementary References

1. Koschmann C, et al (2018) Clinically Integrated Sequencing Alters Therapy in Children and Young Adults With High-Risk Glial Brain Tumors. JCO Precis Oncol 2:PO.17.00133. doi: 10.1200/po.17.00133

2. Miklja Z, et al (2019) Molecular profiling and targeted therapy in pediatric gliomas: review and consensus recommendations. Neuro Oncol 21:968–980. doi: 10.1093/neuonc/noz022

3. Sturm D, et al (2023) Multiomic neuropathology improves diagnostic accuracy in pediatric neuro-oncology. Nat Med 29:917–926. doi: 10.1038/s41591-023-02255-1
